# Nano-rings with a handle – Synthesis of substituted cycloparaphenylenes

**DOI:** 10.3762/bjnano.5.145

**Published:** 2014-08-20

**Authors:** Anne-Florence Tran-Van, Hermann A Wegner

**Affiliations:** 1 Department of Chemistry, Basel University, St Johanns-Ring 19, CH-4056 Basel, Switzerland; 2 Institut für Organische Chemie, Justus-Liebig-Universität, Heinrich-Buff-Ring 62, D-35392 Giessen, Deutschland

**Keywords:** aromatic compounds, carbon nanotubes, cycloparaphenylene, organic synthesis

## Abstract

The research of cycloparaphenylenes (CPPs), the smallest armchair carbon nanotube, has been a quest for the past decades which experienced a revival in 2008 when the first synthesis was achieved. Since then CPPs with various ring sizes have been realized. The incorporation of substituents and the synthesis of CPPs with building blocks different from phenyl rings bear challenges of their own. Such structures, however, are highly interesting, as they allow for an incorporation of CPPs as defined nano-objects for other applications. Therefore, this review provides a status report about the current efforts in synthesizing CPPs beyond the parent unsubstituted oligo-phenylene structure.

## Introduction

Carbon is for organic chemists the essential building block. Besides graphite and diamond scientists have discovered new carbon allotropes such as fullerene [1], graphene [2] and carbon nanotubes (CNTs) [3]. Research on these materials has been originally conducted by physicists. Also, the preparation methods relied on physical processes [4–5]. In the past decade the field is also more and more a playground for organic chemists as these structures are great challenges for modern organic synthesis. In 2002 the first rational synthesis of C_60_ was presented showing the possibility to control each atom in the final target [6]. The choice of suitable synthetic methods is critical to cope with the strain as well as the lack of functional groups in the target molecule. For CNTs a selective synthesis, which controls all structural parameters, is of special interest as they determine the properties and, finally, the field of application [7–10]. For armchair carbon nanotubes, cycloparaphenylenes (CPPs) have been designed as potential template or building block for a bottom-up approach [11–13]. Since 2008 several groups have presented successful syntheses of CPPs with different diameters and their properties were studied [14–24]. The main challenge of such a synthesis is, similar to C_60_, to overcome the high strain energy. To circumvent this, different strategies have been developed. Bertozzi and Jasti used a bent cyclohexadiene that can be converted to a fully aromatic benzene unit [25–32]. Itami utilized a similar L-shaped building block based on cyclohexane [33–38]. Yamago [39–42] relied on a square-shaped tetranuclear platinum complex originally developed by Bäuerle [43] for the synthesis of macrocylic oligothiophenes. In all these methods a macrocyclic precursor is reaching full conjugation in the last step of the synthesis. The strain in a CPP is mainly due to the deviation of the phenyl rings from planarity. However, the phenyl units in all CPPs that were synthesized so far are not fully conjugated due to a torsion between the phenyl moieties to overcome the steric interactions of the protons in *ortho*-position. This effect has to be paid for through an increased energy to deviate the phenyl units from planarity. The incorporation of substituents into CPPs presents therefore a special synthetic challenge as it further increases the torsion angle and, hence, the strain energy. The installment of functionalities, however, is crucial for the application of CPPs. Therefore, this review gathers current endeavors in this direction and discusses the different strategies leading to these new classes of CPP molecules.

## Review

Different types of substitutions have been incorporated in recent syntheses of CPPs. Classical CPP syntheses have opened the way to make phenyl-substituted CPPs, acene-containing CPPs, polycyclic CPPs as well as heteroatom-containing CPPs.

### Phenyl- and alkyl-substituted cycloparaphenylenes

**Scheme 1:**
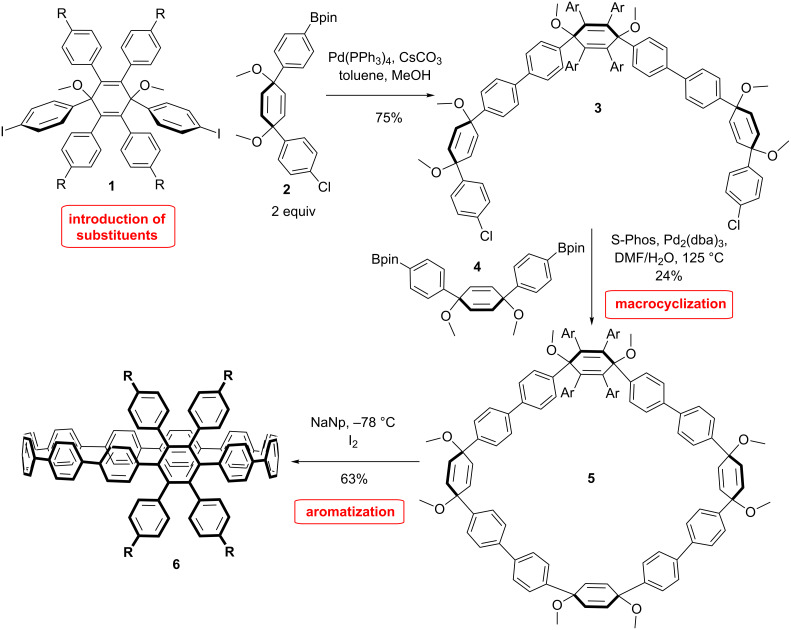
Synthesis of a tetraphenyl-substituted CPP by Jasti et al.

The synthesis of phenyl-substituted CPPs can be performed by using similar strategies as for the unsubstituted CPPs. For instance Jasti et al. applied their strategy to synthesize a tetraphenyl-substituted CPP as a model molecule for the synthesis of larger nanobelts via cyclodehydrogenation reaction (Scheme 1) [44]. The preparation of the modified building block was performed with 1-bromo-4-*n-*butylphenyl that was transformed to the boronic acid. This compound was then coupled with tetrabromoquinone via Suzuki coupling and treated with DDQ to furnish the tetraphenylbenzoquinone. The addition of lithiated phenyl iodide gave the *syn*-diol after separation. The compound was subsequently methylated. They presented a multigram synthesis of this very useful building block **1**, which was coupled via Suzuki reaction with two equivalents of the boronic ester building block **2**. The macrocyclization was again achieved through Suzuki coupling with building block **4** in 24% yield. Finally, the aromatization reaction was conducted by using sodium naphthalenide affording the substituted [12]CPP **6** in 63% yield (Scheme 1).

**Scheme 2:**
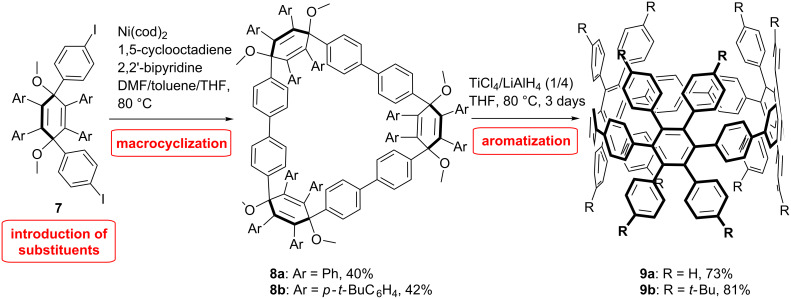
Synthesis of tetraphenyl substituted CPP by Müllen et al.

With the same strategy dodecaphenyl-substituted [9]CPP **9** was prepared by Müllen et al. (Scheme 2) [45]. A tetraphenyl-substituted cyclohexadiene building block **7** was combined via nickel-mediated Yamamoto coupling reaction, and was consequently aromatized through reductive aromatization mediated by the use of low-valent titanium to produce the substituted [9]CPP in even better yields (73–81%) compared to Jasti. The aim of this synthesis was to achieve the synthesis of [3]cyclohexabenzocoronene ([3]CHBC) through cyclodehydrogenation reactions. Indeed, this would be the first three dimensional hexabenzocoronene (HBC) molecule. HBCs are interesting because of their supramolecular behavior and electronical properties [46–48]. However, the cyclodehydrogenation reaction on this molecule proved to be quite challenging due to ring strain and led to a complex mixture of partially dehydrogenated and chlorinated products. A clear evidence for the formation of [3]CHBC was not obtained.

DFT calculations showed that the molecule is *C*_3_-symmetric with a twisted CPP core and a diameter of 12.2 Å in the gas phase. In the calculated structures the dihedral angle is 51.2° between substituted phenyls and unsubstituted phenyls and 78.7° between two unsubstituted phenyl rings. Therefore, π-conjugation is expected to be reduced, which corresponds to the observed shorter absorption and emission wavelengths compared to the unsubstituted [9]CPP. However, the X-ray structures differ from the computationally calculated structures with *C*_1_-symmetry; the phenyl ring fills the cavity due to crystal packing forces, and the ring exhibits an ellipsoidal shape. The ring strain is calculated by using the homodesmotic reaction method to 93.9 kcal/mol. This is 28 kcal/mol more than for the corresponding unsubstituted [9]CPP, and closer to that of the [6]CPP. The NMR study showed that the rotational speed of each phenyl is fast, even at low temperatures, as broadening was only observed at −90 °C.

**Scheme 3:**
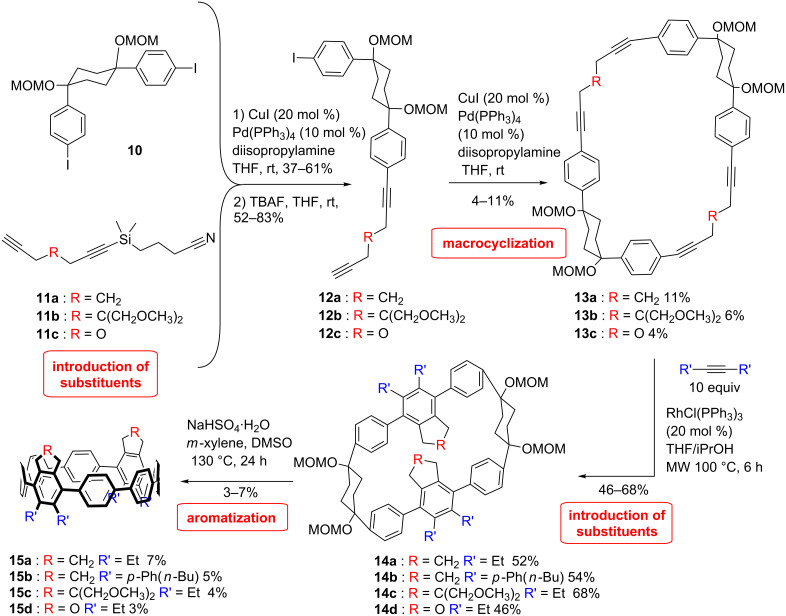
Synthesis of substituted CPPs by Wegner et al.

A different strategy was developed by Wegner et al. (Scheme 3) [49]. Initially, it was planned to utilize the [2 + 2 + 2] cycloaddition reaction to build the CPP scaffold by taking advantage of the aromaticity gained in the process to overcome the ring strain. However, studies on a model system revealed that under the conditions of the [2 + 2 + 2] cycloaddition reaction the reaction pathways deviate in strained systems to a formal [2 + 1 + 2 + 1] cycloaddition reaction [50]. Therefore, a building block was devised that combines a cyclohexane moiety, similar to that of Itami, and a diyne building block precursor for the cycloaddition reaction. With this strategy a modular access to substituted CPPs is provided, which differs from all other approaches in so far as the substituents are introduced after the macrocyclization and not before, which allows for a higher flexibility. The building block **12** was obtained by combining the L-shaped unit **10** with a protected diyne **11** via Sonogashira reaction and subsequent deprotection. Macrocyclization of **12** under Sonogashira reaction conditions provided the macrocyclic dimer **13**. The [2 + 2 + 2] cycloaddition was then performed on the macrocyclic precursor yielding variously substituted macrocycles in 46–52% yield. Finally, differently substituted [8]CPP were obtained through an aromatization reaction of the corresponding precursor **14** (Scheme 3). The low yield of the last step is attributed to the high strain energy to overcome.

Whereas the diameters of substituted rings are comparable to the diameters of unsubstituted [8]CPP, the dihedral angles between the substituted phenyl ring and the neighbouring rings are larger than for two unsubstituted rings, which exhibit a similar angle as in the unsubstituted CPP. Therefore, the conjugation in the substituted CPP is expected to be even less dominant than that in the unsubstituted CPP, which is again represented in the optical properties. The solid-state packing structure shows pairs of substituted CPP molecules arranged parallel to each other in a basket weave motif. Strain energies were calculated by using a different method than the homodesmotic reaction method described in previous publications. Indeed, in this case, the corresponding linear CPP fragments were placed in a repeating supercell. Strain energies between 51.1–73.6 kcal/mol were obtained. The ring strain can vary according to the substitution by around 11 kcal/mol from the strain energy of [8]CPP calculated with the same method (62 kcal/mol).

### Nanorings with inserted acene units

**Scheme 4:**
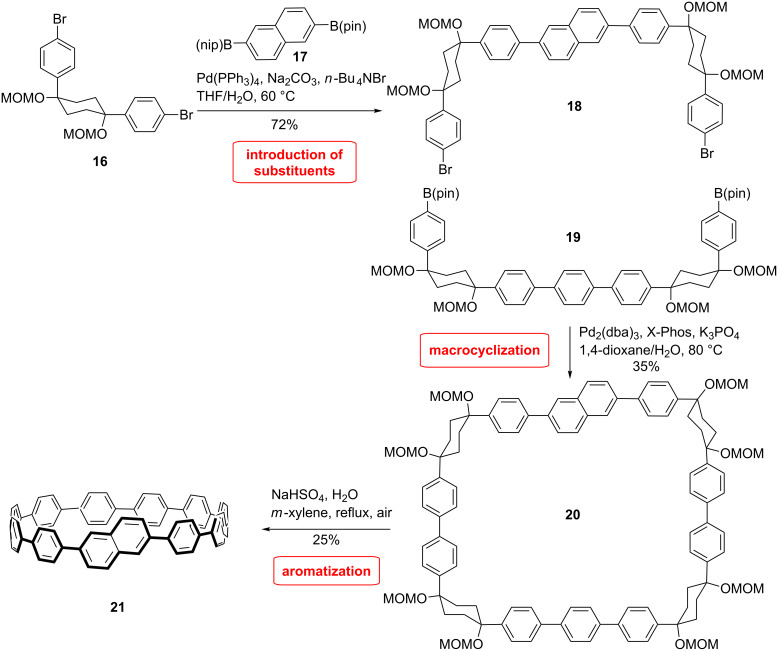
Synthesis of acene inserted CPP by Itami et al.

One of the benefits of applying CPPs as templates for the preparation of CNTs is the possibility to control the chirality of the CNT by incorporating the desired chirality into the precursor. The Itami group applied their concept for the synthesis of the shortest chiral CNTs by inserting an acene unit in a CPP structure (Scheme 4) [51]. As a particular example they synthesized cyclo[13]*para-*phenylen-2,6-naphthylene ([13]CPPN). With this approach it is possible to synthesize all chiral nanohoops by including the appropriate acene. The synthesis follows the classical strategy developed by the group (Scheme 4). The required L-shaped building block **16** was synthesized according to a previously developed methodology [37]. Two of these building blocks were coupled with 2,6-diborylated naphthalene **17** to give the first U-shaped building block **18**. The second U-shaped unit **19** was synthesized accordingly by Suzuki coupling of diborylated benzene with two equivalents of L-shaped building block **16**. The resulting compound was then borylated. The two units **18** and **19** were combined by Suzuki coupling to form the square-shaped macrocycle **20** in 35% yield. The CPP precursor was subsequently aromatized by a treatment with sodium bisulfate to furnish the desired [13]CPPN in 25% yield.

[13]CPPN **21** presents a helical chirality, although racemization occurs by rotation of the acene unit around the connecting bonds to the rest of the ring. Calculations showed that the molecule is racemizing rapidly at ambient temperature (8.4 kcal/mol). They also studied the racemization of other chiral nanorings to study the impact of the ring size. The racemization barrier increases as the ring diameter decreases and also as the acene unit becomes larger, which can be explained by the increase of ring strain.

**Scheme 5:**
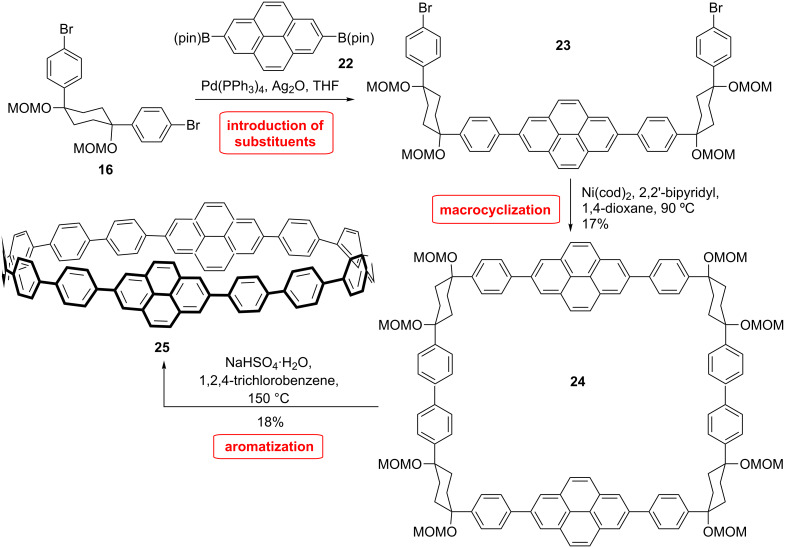
Synthesis of a pyrene-inserted CPP by Itami et al.

Furthermore, a synthesis of a pyrene-inserted CPP was developed by the Itami group (Scheme 5) [52]. This molecule represents a CPP that was benzannulated and π-extended at the bay-region by converting two biphenyl units into a pyrene unit. Pyrene derivatives show interesting photophysical properties like long fluorescence life time and excimer emission. Therefore, it was of interest to see if these features appear in the CPPs containing pyrene units. The preparation of cycloparaphenylene-2,7-pyridylene (CPPyr) is described in Scheme 5. The synthesis relies on the same strategy as that for the corresponding non-annelated CPP. It uses the L-shaped building block **16** and the pyrene unit is incorporated via Suzuki coupling of the diborylated 2,7-pyrene **22** to access the U-shaped unit **23**. The two units were coupled using a nickel catalyst to give the macrocyclic dimer **24** in 17% yield. Aromatization of this CPP precursor was achieved by sodium bisulfate in 1,2,4-trichlorobenzene to give the desired [12,2]CPPyr **25**. Notably, the resulting pyrene product was soluble only in chlorinated solvents in contrast to the generally easily soluble CPPs.

The UV–vis absorption spectrum can be explained by a combination of the spectra of pyrene and [16]CPP, as a result of the poor conjugation between paraphenylene and 2,7-pyrenylene. The emission maximum appears at 430 nm similar to [12]–[18]CPPs. However, the quantum yield is found to be much smaller with 0.21 in comparison to [9]–[16]CPPs (0.73–0.90) [15]. The fluorescence life time is larger than that of other CPPs with 25.6 ns (10.6 ns for [12]CPP), which is in accordance with the long fluorescence life time of pyrenes. A computational study showed that HOMO−1 HOMO−2, LUMO+1 LUMO+2 are degenerated and localized on the two pyrene moieties and have a similar shape as the HOMO and LUMO of pyrene explaining the additivity of the UV spectra of [16]CPP and pyrene. The shape and energy of HOMO and LUMO are similar to those of [16]CPP. Therefore, the emission is mostly dependent on the CPP structure. Recently, Yamago and coworkers also reported the synthesis of a [4]cyclo-2,7-pyrenylene ([4]CPY) by using their method with a square-like tetra-Pt-complex [53]. DFT-calculations as well as electrochemical analysis revealed a strong resemblance with [8]CPP.

**Scheme 6:**
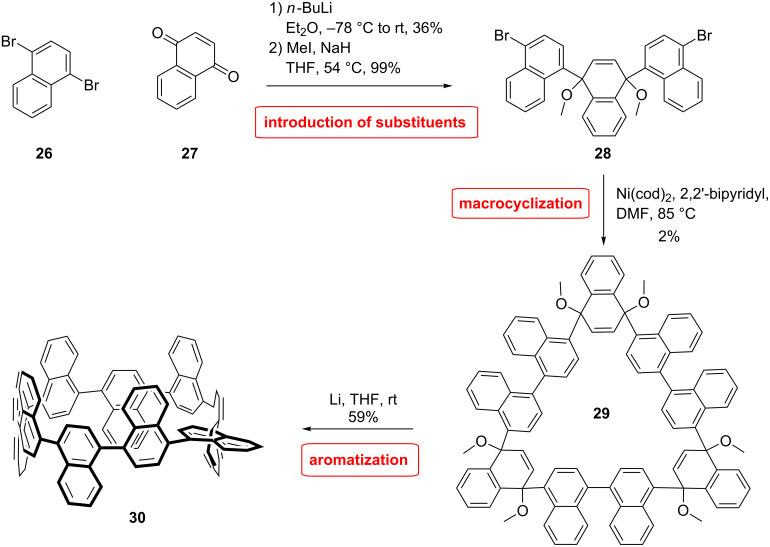
Synthesis of a [9]cyclonaphthylene nanoring by Itami et al.

During the evolution of larger π-extended nanobelts, Itami applied the cyclohexadiene strategy that was initially presented by Jasti et al. with a modified naphthalene derivative to synthesize a cyclonaphthylene nanoring (CN) (Scheme 6) [54]. The building block was synthesized by addition of mono-lithiated 1,4-dibromonaphthalene (**26**) on 1,4-naphthoquinone (**27**), providing the desired *cis*-diol in 36% yield after separation. The diol was further methylated by using MeI and NaH as base. They combined the designed L-shaped naphthylene modified building block **28** in a nickel-mediated shotgun macrocyclization producing the trimer **29** in 2% yield. Final aromatization under reductive condition afforded desired π-extended [9]CN **30** in 59% yield [55].

The NMR spectrum of [9]CN is differentiated into numerous peaks that coalesce into three signals when heated to 150 °C. Therefore, the molecule has a low symmetry and dynamic conformational changes take place slowly on the NMR time scale at room temperature. Because of steric reasons, the dihedral angles between the naphthalene rings are around 60°, which is twice as much as in [9]CPP. [9]CPP as well as [9]CN are odd numbered which means it is not possible that the phenyl or naphthyl rings arrange in an alternated twisted structure. Therefore, a helical moiety has to be incorporated in the ring. In the case of [9]CN one of the naphthalene rings is parallel to the plane of the macrocyle. As a result, the molecule is chiral. The racemization was studied by computational models and compared with the rotation of 1,1’-binaphtyl moieties, observing an effect of the ring strain on the rotation mode. Compared to 1,1’-binaphthyl the interaction of the C8–H/C8’–H is less important due to the ring curvature. The maximum of absorption (378 nm) is shifted towards longer wavelengths than for the [9]CPP (340 nm) which can be attributed to the extension of the π-system. Fluorescence emission occurs at 491 nm which is similar to the corresponding CPP however with a quantum yield lowered by a factor of two [15].

**Scheme 7:**
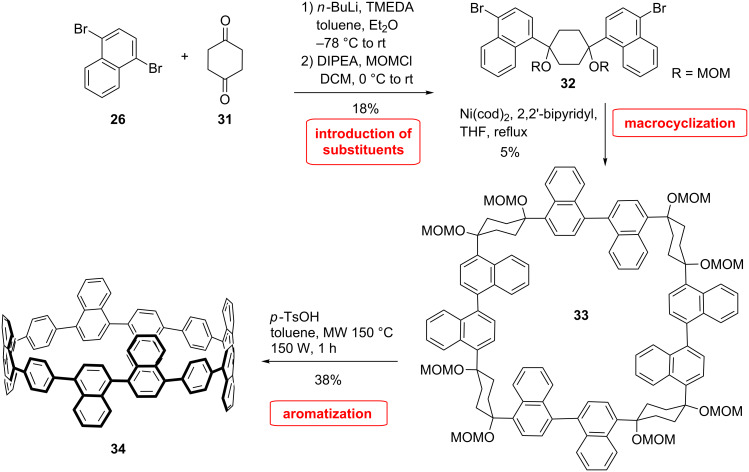
Synthesis of a naphthylene-containing CPP by Swager and Batson.

Another naphthalene CPP has been synthesized by Batson and Swager [55], who used a similar strategy as the Itami group. They choose to use a benzene spacer to isolate 1,1’-binaphthyl units in order to facilitate the Scholl reaction to form perylene units (Scheme 7). Molecule **34** constitutes a good model for probing reaction conditions, which would be usable in the synthesis of larger nanobelts through cyclodehydrogenation.

**Scheme 8:**
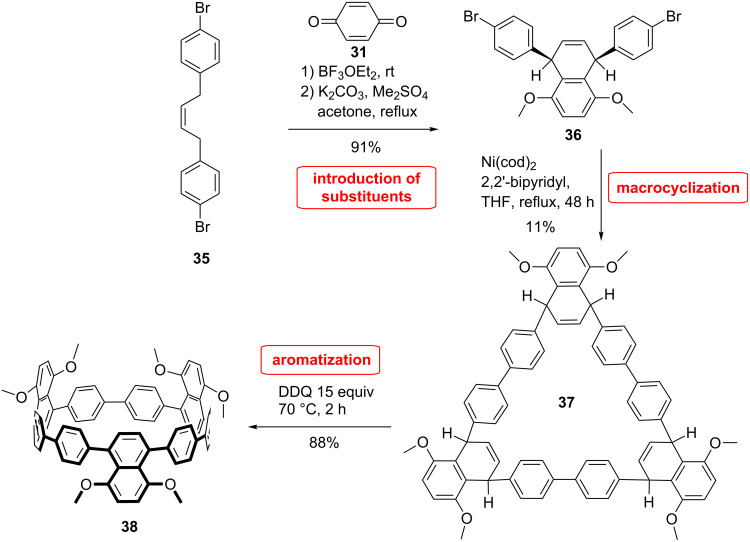
Synthesis of functionalized cycloparaphenylenes by Wang et al.

The group of Wang [56] explored a different strategy to integrate a substituted naphthylene unit into CPP (Scheme 8). The square shaped subtituent was synthesized via Diels–Alder reaction of **35** and **31** selectively affording the *cis* product **36** after methylation. This building block was further submitted to macrocyclization using nickel-catalyzed homocoupling initially developed by Itami et al. The *syn* and *anti* trimers were isolated in 11% yield. Dimers were also isolated. The aromatization of **37** was performed by using DDQ yielding the corresponding [9]CPP with three 5,8-dimethoxynaphth-1,4-diyl units in 88% yield. However, the aromatization of the dimer precursor for the [6]CPP was not successful due to larger strain energy. The NMR study of **38** suggests that the phenyl units undergo fast rotation as only one set of signals was observed.

### CPP dimers and other architectures

**Scheme 9:**
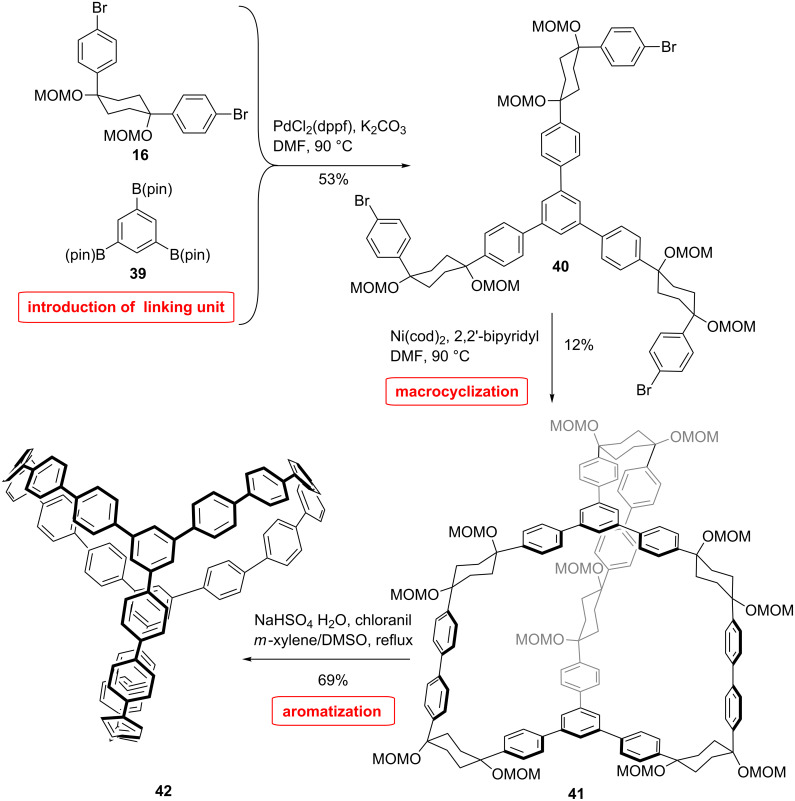
Synthesis of a CNT junction by Itami et al.

The structure of *para*-connected CPP resembles that of linear CNTs. The incorporation of other connectivities such as *meta* allows for the assembly of other complex geometries. The synthesis of a nanocage of interlinked CPP was performed by Itami et al. (Scheme 9) [57]. They used their L-shaped cyclohexane building block **16** coupled to a 1,3,5-triborylbenzene **39** as three-way unit. Suzuki coupling and macrocyclization afforded the precursor **41**. Aromatization of this cage precursor produced the beautiful conjugated nanocage molecule **42**, which could serve as template for a CNT junction.

Calculations show that the molecule has a *D*_3_ symmetry, the tri-substituted benzene units are staggered by 12.5°, and the distance between these two connection units is 18.4 Å, which lies between the diameter of [13]CPP and [14]CPP. The strain energy was calculated through the homodesmotic reaction method to 67.2 kcal/mol.

**Scheme 10:**
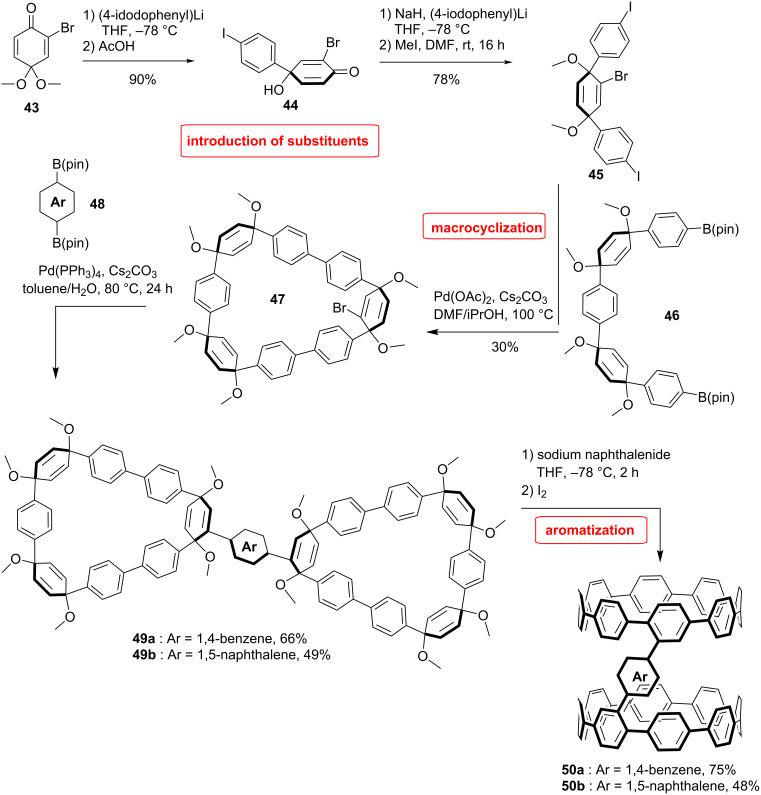
Synthesis of CPP dimers by Jasti et al.

Dimers of CPPs were prepared in the Jasti group by using a bromo-substituted CPP precursor (Scheme 10) [58]. The synthesis relied on a brominated cyclohexadiene building block **45**. This entity was then inserted in compound **46**, which was synthesized by a succession of Suzuki–Miyaura coupling reactions. With the bromo-functionalized macrocycle **47** in hand they performed the dimerization by coupling two macrocycles to an arene unit, either 1,4-benzene boronic ester (66%) or 1,5-naphtalene boronic ester (49%). The dimeric macrocycles were then aromatized under reductive conditions to obtain the corresponding CPP dimers **50** in 75% and 48% yield for the benzene and naphthalene junction, respectively. The molecules were rather insoluble, which is in sharp contrast to the monomeric CPP which is readily soluble in organic solvents.

There are two conformers possible for the molecule: the *cis* conformer, in which the two rings are on top of each other like in a nanotube, and the *trans* conformer, in which the rings are far from each other. The *cis* conformer could serve as a starting point to construct a longer nanotube by closing more carbon–carbon bonds via Scholl reaction. Switching between the two configurations allows for interesting applications in host–guest chemistry. In the solid state, the *trans* structure was calculated to be lower in energy than the *cis* isomer. However, the dynamic change from *trans* to *cis* conformation was studied by DFT calculation and the energy barrier was found to be 13 kcal/mol and 20 kcal/mol for the reverse. The path with the lowest energy implies the flipping of one CPP ring over the other and not a simple rotation around the aryl linking unit. In this dynamic study the *cis* configuration was found to be the lowest in energy in the gas phase due to increased van der Waals interactions between the two CPP rings. Studies including solvent effects did not change the relative stability of the conformers compared to the gas phase calculations.

**Scheme 11:**
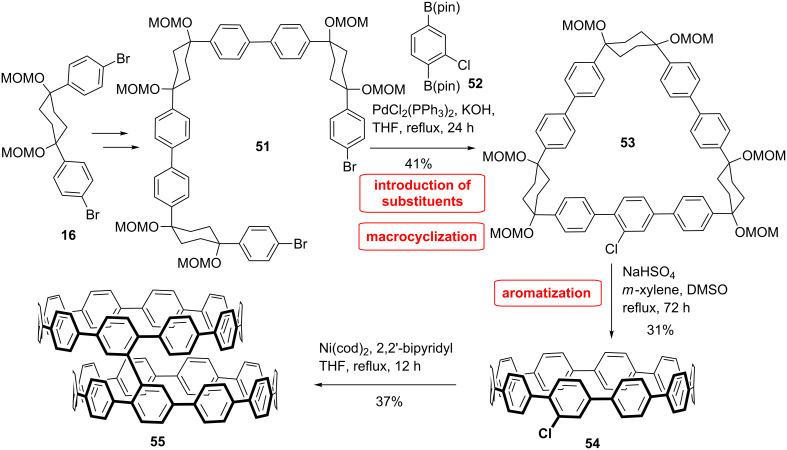
Synthesis of a CPP dimer by Itami et al.

A directly linked CPP dimer has been synthesized by the group of Itami (Scheme 11) [59]. The synthesis relied again on the L-shaped building block **16** combined with a chlorobenzene unit **52**. This way, they accessed the first monohalogenated [10]CPP. This molecule offers various possibilities for further functionalization. They successfully managed the dimerization of the chloro[10]CPP **54** through Yamamoto coupling. The isomerization between the *cis* and *trans* configurations was studied by DFT calculations. Here again the most stable conformation is the *cis* form. The results show that the transition takes place through rotation of one of the CPP rings around the connected benzene ring with a barrier of 8.9 kcal/mol indicating a fast isomerisation process at room temperature.

**Scheme 12:**
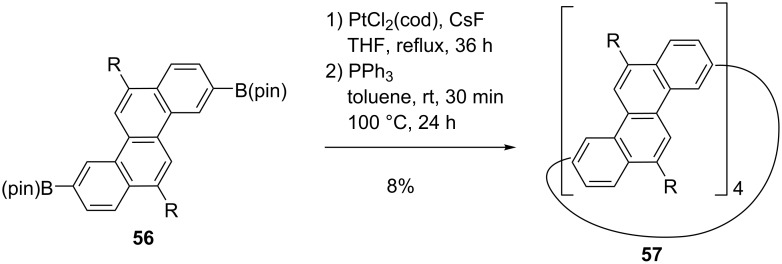
Synthesis of a zig-zag CNT fragment by Isobe et al.

Another synthesis of acene-based rings has being developed by the Isobe group (Scheme 12). Their work afforded different fragments of nanotubes [60–61], for instance, a fragment of a zig-zag CNT was targeted [60]. The shortest zig-zag nanotube is the cyclacene fragment. However, the synthesis of this molecule remains challenging, mainly because of the reactive edges. As an alternative, Isobe et al. prepared a fragment of zig-zag CNT by using chrysenylene building blocks coupled via platinum complexation and reductive elimination, featuring the strategy also applied by Yamago [60]. Surprisingly, only one diastereomer was obtained. Different atropisomers are possible depending on the orientation of the chrysene units. The two optically active enantiomers formed by this reaction could be separated.

The isolation of the enantiomers shows the strain induced atropisomerism. There is a strong rotational restriction at the single bond linkages maintaining the tube-like shape. Moreover, the isomerization process could not be observed even at high temperatures showing a large rotation barrier and constriction at the single bond linkage. The solid state structure of the racemate presents a thread-in-bead columnar assembly with the hexyl chain of one enantiomer incorporated into the center of the other enantiomer forming pairs of enantiomers entangled together. Several pairs arrange in a tubular manner by incorporating hexyl chains of other pairs.

### Heteroatomic cycloparaphenylenes

**Scheme 13:**
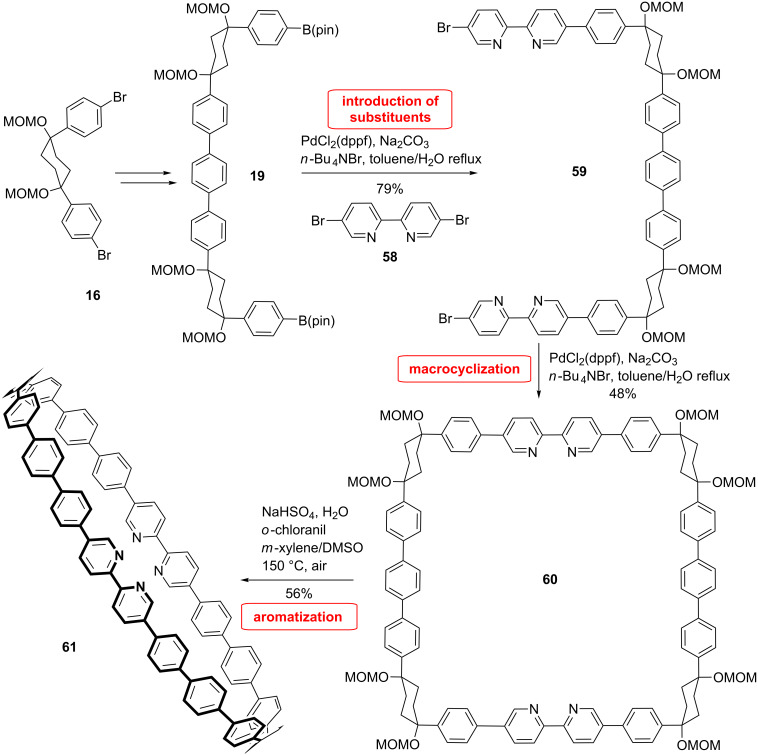
Synthesis of a CPP with an inserted bipyridine by Itami et al.

With all the strategies available, it is also possible to synthesize CPPs which contain heteroatoms, such as the nitrogen-containing CPP synthesized by Itami et al. by using bipyridine as a linear building block [62]. Such heteroatom-containing rings could pave the way to novel properties of CPPs through supramolecular assemblies, complex formation, or acid–base modifications and therefore offer access to new applications. The strategy for this synthesis relied on the same building block used by the Itami group for the synthesis of the parent CPP the only exception being the bipyridine building block which was inserted on two sides of compound **19** (Scheme 13). The bipyridine **58** was then again coupled with **19** in a Suzuki reaction to give the macrocyclic precursor **59** in 48% yield. Finally, the CPP **61** was obtained in 56% yield by an aromatization reaction mediated by sodium bisulfate in the presence of chloranil.

Whereas the optical properties of **61** resemble those of large CPPs, a strong dependence on the pH value was observed. The absorption and emission spectra show broadening as well as red shift. This can be due a charge transfer excited state upon protonation of the bipyridine units. When the solution was neutralized, the previously observed absorption and fluorescence spectra were reproduced showing the reversibility of the process.

## Conclusion

Different syntheses of CPPs of different diameters have been established. Based on these strategies a variety of substituted and non-phenyl containing CPPs have been prepared. All examples have the key steps of introducing the substituents, macrocyclization and finally aromatization. The possibility to add different substituents at a late stage in the synthesis is especially attractive. The incorporation of further functionalities into CPPs opens the way for the synthesis of larger nanobelts and other complex molecular architecture promising more and more creative new CPP-based structures in the near future.
